# Dual inhibition of PCDH9 expression by miR-215-5p up-regulation in gliomas

**DOI:** 10.18632/oncotarget.14396

**Published:** 2016-12-31

**Authors:** Chunlin Wang, Qi Chen, Shu Li, Shiting Li, Zhenyu Zhao, Hongliang Gao, Xiaoqiang Wang, Bin Li, Wenchuan Zhang, Yan Yuan, Linzhao Ming, Hua He, Bangbao Tao, Jun Zhong

**Affiliations:** ^1^ Department of Neurosurgery, The 105th Hospital of PLA, Hefei, Anhui 230000, China; ^2^ Department of Anesthesiology, Xinhua Hospital, School of Medicine, Shanghai Jiaotong University, Shanghai 200003, China; ^3^ Department of Pathophysiology, Wannan Medical College, Wuhu 241002, China; Department of Neurosurgery, Wuxi Second People’s Hospital, Wuxi, Jiangsu, 214002, China; ^4^ Department of Neurosurgery, Xinhua Hospital, School of Medicine, Shanghai Jiaotong University, Shanghai 200003, China; ^5^ Department of Neurosurgery, Chinese PLA General Hospital, Beijing 100003, China; ^6^ Department of Neurosurgery, Changzheng Hospital, The Second Hospital affiliated with The Second Military Medical University, Shanghai 200003, China

**Keywords:** PCDH9, miR-215-5p, glioma, miRNA, integrative analysis

## Abstract

The clinical prognosis of malignant gliomas is poor and PCDH9 down-regulation is strongly associated with its poor prognosis. But the mechanism of PCDH9 down-regulation is unknown. Abnormal miRNAs profiles regulate tumor phenotypes through inhibiting their target genes and miRNAs could inhibit target genes more efficiently by binding to both the promoter and 3′UTR of target genes. In this study, to search the dual inhibitory miRNAs which suppress PCDH9 expression in gliomas, we performed an integrative analysis of databases including miRDB, TargetScan, microPIR and miRCancer. We identified three candidate miRNAs which were predicted to bind both the promoter and 3′UTR of PCDH9 and up-regulated in gliomas. Then, we validated miR-215-5p up-regulation and PCDH9 down-regulation in glioma samples and demonstrated that miR-215-5p could inhibit the mRNA and protein levels of PCDH9 in glioma cell lines by targeting its promoter and 3′ UTR at the same time. Moreover, miR-215-5p could increase glioma cell proliferation, clone formation, *in-vitro* migration and reduce apoptosis via inhibiting PCDH9 expression. Our study provides evidence for a novel dual inhibition of PCDH9 by miR-215-5p in gliomas and suggests that miR-215-5p might be a therapeutic target for the treatment of gliomas.

## INTRODUCTION

Malignant gliomas make up about 70% of all malignant brain tumors [1]. Although important progress has been made in understanding its molecular pathogenesis, the prognosis for patients with high-grade gliomas is generally poor [2, 3]. An un-biased integrative study combining chromosomal alterations and gene expression data identifies PCDH9 as a potential tumor suppressor in gliomas [4]. PCDH9 is a member of the protocadherin family and cadherin superfamily. The general function of cadherins is to mediate cell-cell adhesion and recognition in the presence of calcium [5]. Further studies show that PCDH9 down-regulation is associated with higher histological grade and poor prognosis [6]. PCDH9 knock-down exacerbates the tumor phenotypes in glioma cell lines [7]. Given the important roles of PCDH9 in gliomas, the regulatory mechanism of PCDH9 expression is critical but almost unknown.

Studies have shown that miRNAs regulate one third of the human genes [8] and abnormal miRNA profiles regulate tumor phenotypes through inhibiting their target genes [9, 10]. Thus, abnormal microRNAs might induce PCDH9 down-regulation in gliomas. Most miRNAs bind to the 3′ un-translated region (UTR) of target genes [11] while some miRNAs bind to the promoter region [12–14]. In the past, it’s unknown if one single miRNA could bind to both the 3′ UTR and promoter of the same target gene. Recently, a study shows that miR-552 could inhibit cytochrome P450 2E1 more efficiently by this novel dual inhibition in hepatoma cell lines [15]. Thus, there might to be a unique subgroup of miRNAs with dual inhibitory effects on their target genes. Targeting these miRNAs might achieve higher therapeutic efficacy and minimize off-target effects.

In this study, we first predicted the miRNAs which might bind to both the promoter and 3′UTR of PCDH9 by integrative analysis and then measured the expression levels of candidate miRNAs and PCDH9 in glioma samples. We found a strong association between miR-215-5p up-regulation and PCDH9 down-regulation in gliomas. We further validated that miR-215-5p could inhibit PCDH9 expression by binding its promoter and 3′UTR in glioma cell lines. Moreover, miR-215-5p promotes aggressive phenotypes of glioma cell lines through inhibiting PCDH9 expression. Our study provides evidence for a novel dual inhibition of PCDH9 by miR-215-5p in gliomas and suggests that miR-215-5p might be a therapeutic target for the treatment of gliomas.

## RESULTS

### Integrative analysis of miRNAs targeting the promoter and 3′ UTR of PCDH9 in human gliomas

**Figure 1:**
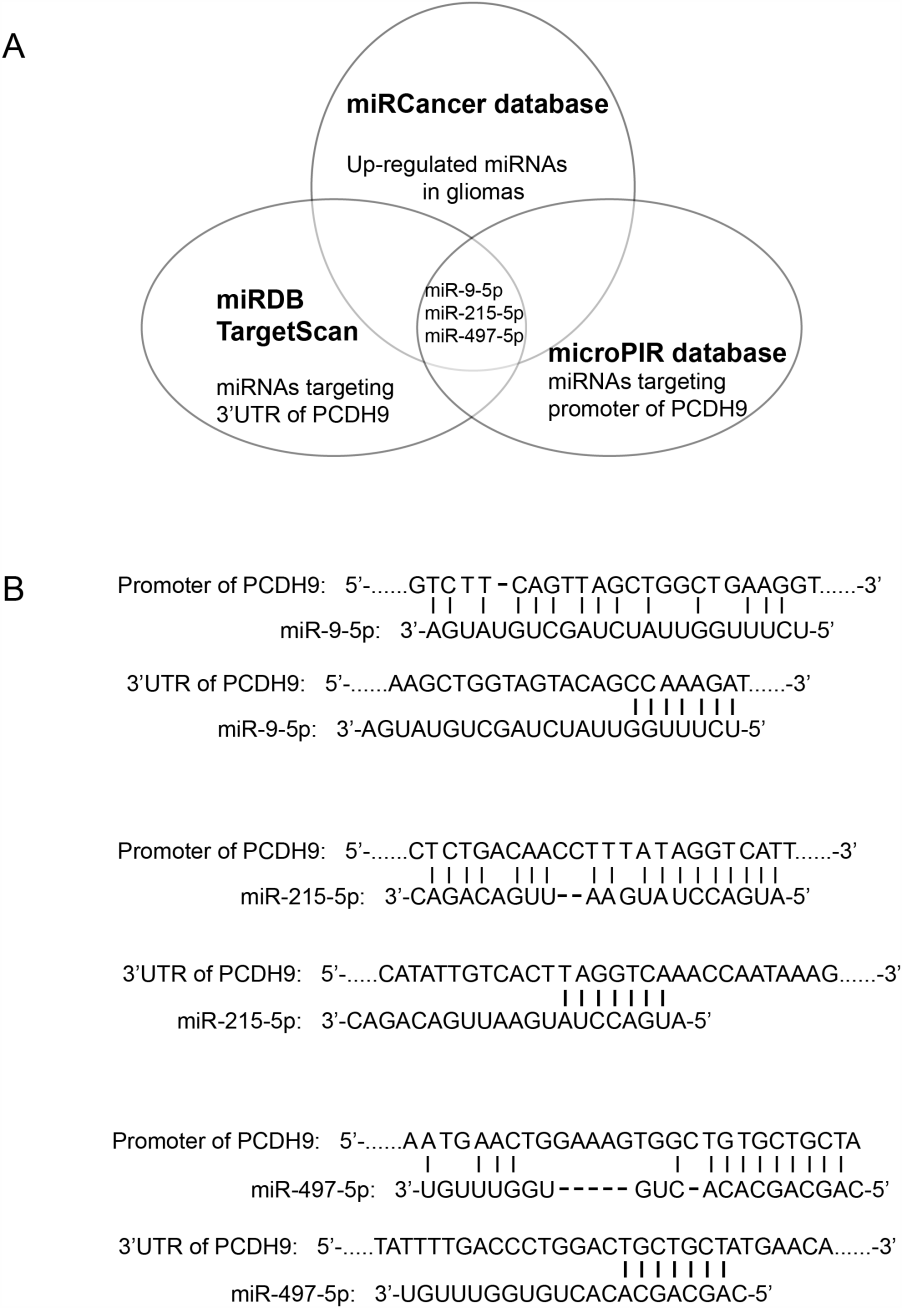
Integrative analysis of miRNAs targeting the promoter and 3′ UTR of PCDH9 in human gliomas **A**. Diagram of predicted miRNAs from indicated miRNA databases. **B**. Complementary pairing of miR-9-5p, miR-215-5p and miR-497-5p to the promoter and 3′ UTR of human PCDH9 gene, respectively.

To identify the potential miRNAs with dual inhibitory effects on PCDH9 expression in gilomas, The miRDB [16] and TargetScan [17] were used to predict the potential miRNAs targeting 3′ UTR of PCDH9. The microPIR database [18] was used to predict the potential miRNAs targeting the promoter of PCDH9. The miRCancer database [19] was used to search the up-regulated miRNAs in gliomas. Then, we compared the three lists of candidate miRNAs from the above data-mining and found a small overlap of three miRNAs, miR-9-5p, miR-215-5p, miR-497-5p (Figure 1A). The potential binding sites of miR-9-5p, miR-215-5p, miR-497-5p in the promoter and 3′ UTR of PCDH9 are shown in Figure 1B.

### The expression levels of miRNAs and PCDH9 in human gliomas

**Figure 2:**
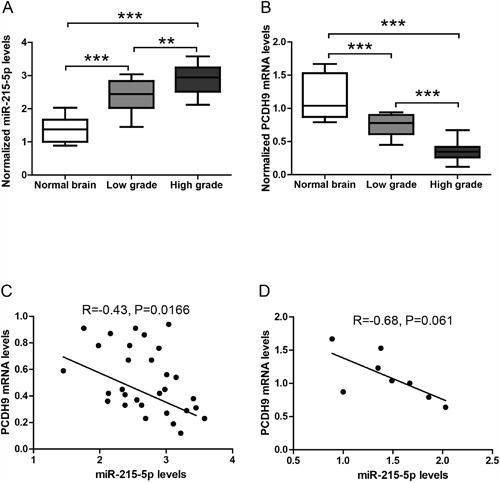
The expression levels of miRNAs and PCDH9 in human gliomas The qRT-PCR results showing the relative miR-215-5p levels **A**. and PCDH9 mRNA levels **B**. in normal brain tissues (n=8), low-grade gliomas (n=12) and high-grade gliomas (n=18). Association analysis of miR-215-5p and PCDH9 mRNA levels in glioma tissues **C**. R=-0.43, P=0.0166 and normal brain tissues **D**. R=-0.68, P=0.061. Data were presented as box-whisker plots. For all, **P<0.01; ***P<0.001.

We measured the expression levels of miR-9-5p, miR-215-5p and miR-497-5p in glioma samples using TaqMan assays. The results showed that miR-215-5p levels in gliomas were up-regulated compared to those in normal brain tissues (Figure 2A). Moreover, miR-215-5p levels in high-grade gliomas were higher than those in low-grade gliomas, suggesting that miR-215-5p plays an important role in glioma progression. In contrast, there was no significant difference in the expression levels of miR-9-5p and miR-497-5p between gliomas and brain normal tissues (data not shown). We also measured PCDH9 mRNA levels in glioma samples. Our previous studies showed down-regulation of PCDH9 protein levels in gliomas [6, 7]. Consistently, PCDH9 mRNA levels in gliomas were down-regulated compared to those in normal brain tissues (Figure 2B). Moreover, PCDH9 mRNA levels in high-grade gliomas were lower than those in low-grade gliomas. We further analyzed the potential association between miR-215-5p and PCDH9 mRNA levels in glioma tissues and normal brain tissues. There was a significant negative correlation between miR-215 and PCDH9 mRNA level in gliomas (R=-0.43, P=0.0166, Figure 2C). In normal brain tissues, there was a clear trend but it did not reach statistical significance (R=-0.68, P=0.061, Figure 2D), possibly due to small sample size. These results from clinical samples provide initial evidence that miR-215-5p might inhibit PCDH9 expression in gliomas.

### The miR-215-5p inhibits PCDH9 expression by targeting its promoter and 3′ UTR

**Figure 3:**
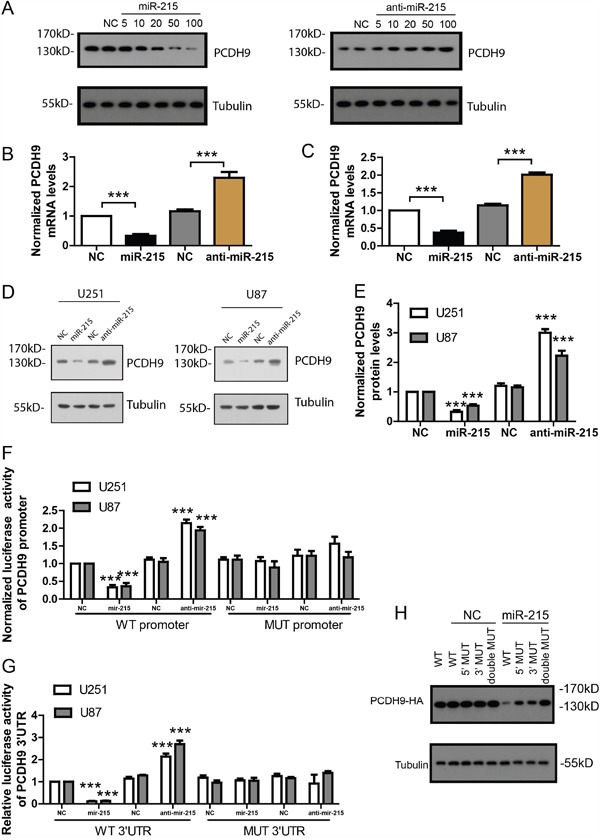
The miR-215-5p inhibits PCDH9 expression by targeting its promoter and 3′ UTR **A**. WB results showing PCDH9 protein levels after over-expression with miR-215-5p mimic or knocking-down with anti-miR-215 inhibitor at the concentration of 5, 10, 20, 50 and100 nM for 48h in U251. The qRT-PCR results showing PCDH9 mRNA levels after over-expression or knocking-down of miR-215-5p in U251 B. n=3 and U87 C. n=3 cell lines. Representative WB results **D**. and its quantification **E**. showing PCDH9 protein levels after over-expression or knocking-down of miR-215-5p in U251 and U87 cell lines. Luciferase reporter assays showing the effects of over-expression or knocking-down of miR-215-5p on the wild-type or mutated promoter **F**. and 3′UTR **G**. of PCDH9, respectively. **H**. U251 cells were transfected with wild-type, promoter-mutated (5′ MUT), 3′UTR-mutated (3′MUT) or double mutated PCDH9-HA vector and miR-215-5p mimic. WB results showing PCDH9-HA protein levels detected by HA antibody. Data were presented as means ± s.e.m. For all, ***P<0.001.

To further confirm if miR-215-5p could inhibit PCDH9 expression, we over-expressed miR-215-5p at the concentration of 5-100 nM in glioma cell line U251. The result showed that miR-215-5p over-expression reduced PCDH9 protein levels in a dose-dependent manner. Consistently, knock-down with miR-215-5p inhibitor increased PCDH9 protein levels in a dose-dependent manner (Figure 3A). The 20 nM concentration of miR-215-5p mimic and inhibitor was used for following experiments. Results of real time-PCR (Figure 3B and 3C) and WB (Figure 3D and 3E) showed that miR-215-5p over-expression reduced the mRNA and protein levels of PCDH9 while miR-215-5p knock-down increased the mRNA and protein levels of PCDH9. To investigate the potential effects of miR-215-5p on the promoter activity of PCDH9, we cloned the PCDH9 promoter into pGL4.10, a luciferase reporter vector. In HEK293 cells transfected with PCDH9 promoter luciferase vector, miR-215-5p mimic greatly suppressed the luciferase activity while miR-215-5p inhibitor increased the luciferase activity (Figure 3F). To investigate the potential effects of miR-215-5p on the 3′ UTR of PCDH9, we cloned the 3′ UTR of PCDH9 into a luciferase reporter vector pMIR. In HEK293 cells transfected with PCDH9 3′ UTR luciferase vector, miR-215-5p mimic greatly suppressed the luciferase activity while miR-215-5p inhibitor increased the luciferase activity (Figure 3G). Once the predicted binding sites in the promoter or 3′ UTR of PCDH9 were mutated, the effects of miR-215-5p mimic or inhibitor were abolished, indicating that the endogenous miR-215-5p could directly target the promoter and 3′ UTR of PCDH9.

To demonstrate the dual inhibition of miR-215-5p on PCDH9 expression, we constructed an HA tagged expression vector with PCDH9 CDS under the control of its own 500bp promoter and 500bp 3′UTR. The PCDH9-HA expression was detected with HA antibody. In U251 transfected with PCDH9-HA vector, miR-215-5p could inhibit PCDH9-HA expression effectively (Figure 3H). When the binding site in the promoter or 3′UTR was mutated, the inhibitory effect of miR-215-5p on PCDH9-HA was only partially impaired. Once the binding sites in the promoter and 3′UTR were both mutated, the inhibitory effect of miR-215-5p was totally abolished. These results suggest that dual inhibition of PCDH9 by miR-215-5p is more effective than targeting the promoter or 3′UTR alone.

### The miR-215-5p promotes glioma phenotypes via inhibiting PCDH9 expression

**Figure 4:**
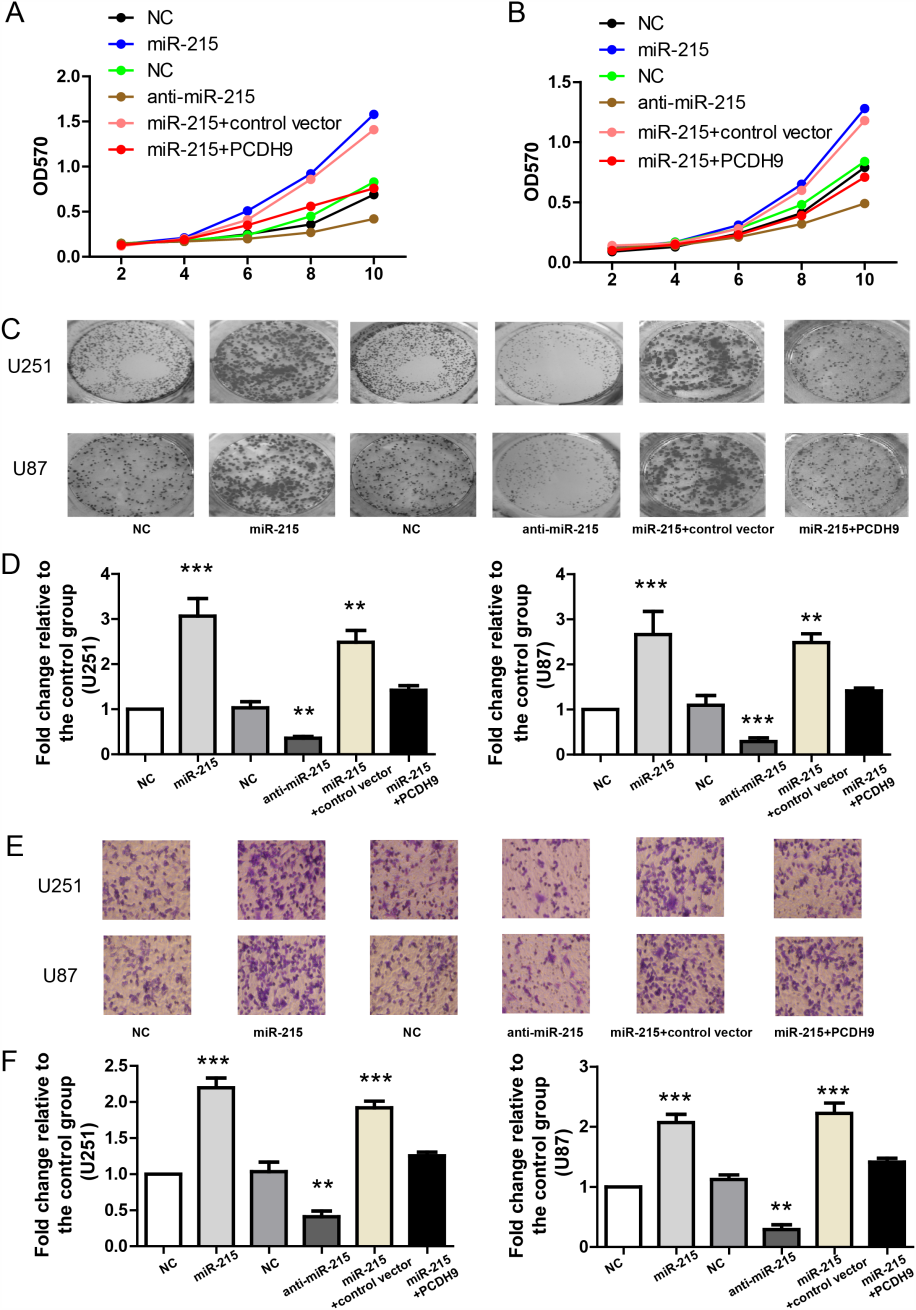
The miR-215-5p promotes glioma phenotypes via inhibiting PCDH9 expression **A**. MTT assays showing the growth curves of U251 (A) n=3 and U87 B. n=3 after transfection with indicated miRNAs or vectors. Representative images of clone formation **C**. and its quantification **D**. showing the clone numbers after transfection with indicated miRNAs or vectors in U251 (n=3) and U87 (n=3) cell lines. Representative images of *in-vitro* migration assays **E**. and its quantification **F**. showing the migrated cells after transfection with indicated miRNAs or vectors in U251 (n=3) and U87 (n=3) cell lines. For all, **P<0.01; ***P<0.001.

As previous study shows that PCDH9 plays important roles in proliferation, tumor formation and apoptosis, we sought to determine if miR-215-5p also promotes these tumor phenotypes via inhibiting PCDH9 expression in two glioma cell lines U251 and U87. First, MTT assay was used to measure the effects of miR-215-5p on cell proliferation in U251 and U87. The results showed that miR-215-5p mimic promoted but miR-215-5p inhibitor suppressed cell proliferation (Figure 4A and 4B). Moreover, PCDH9 over-expression could rescue the stimulatory effects of miR-215-5p mimic. In the clone formation assay, miR-215-5p mimic increased but miR-215-5p inhibitor reduced clone numbers (Figure 4C and 4D). PCDH9 over-expression could rescue the stimulatory effects of miR-215-5p mimic. Similarly, in the *in-vitro* migration assay, miR-215-5p mimic promoted but miR-215-5p inhibitor suppressed cell migration and PCDH9 over-expression could rescue the effects of miR-215-5p mimic (Figure 4E and 4F).

**Figure 5:**
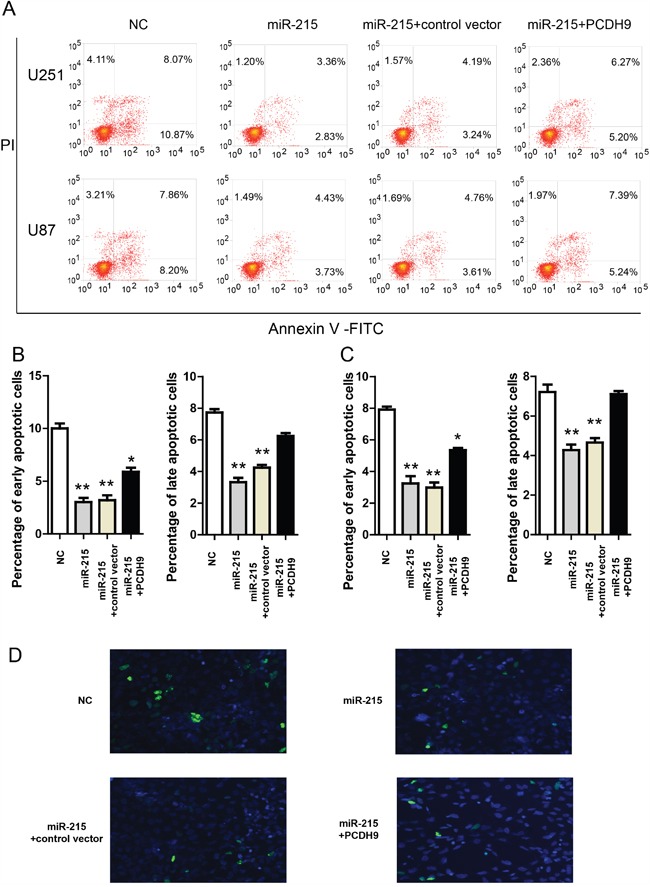
The miR-215-5p reduces glioma apoptosis via inhibiting PCDH9 expression **A**. Representative results of Annexin V-FITC apoptosis assays showing the effects of miR-215-5p mimic and indicated vectors in U251 and U87 cell lines. The quantification of the effects of miR-215-5p mimic and indicated vectors on the percentage of apoptotic cells in U251 B. n=3 and U87 C. n=3 cell lines. **D**. Representative images of TUNNEL staining in U251 cells transfected with miR-215-5p mimic and indicated vectors for 48h. Data were presented as means ± s.e.m. For all, *P<0.05; **P<0.01.

Moreover, in the Annexin V-FITC apoptosis assay, miR-215-5p mimic reduced apoptosis in U251 and U87 (Figure 5A, 5B and 5C). PCDH9 over-expression could rescue the effects of miR-215-5p mimic. Consistently, TUNNEL assay in U251 cells showed that miR-215-5p mimic reduced apoptosis and PCDH9 over-expression could rescue the effects of miR-215-5p mimic (Figure 5D). These results support that miR-215-5p up-regulation promotes aggressive phenotypes of glioma cell lines via inhibiting PCDH9 expression. And this is also consistent with the fact that miR-215-5p level was highest in high-grade gliomas (Figure 2A).

## DISCUSSION

PCDH9 plays important roles in many types of cancer [20] and its down-regulation is also found in gastric cancer [21] and hepatocellular carcinoma [22]. In addition, PCDH9 might be a drug target for cancer treatment [23, 24]. Thus, insights from PCDH9 regulation might bring new opportunity for glioma treatment.

Previous studies show that miR-215-5p plays important roles in osteosarcoma [25], colon cancer [25], renal cell carcinoma [26, 27] and gastric cancer [28–30]. Two recent studies reveal that miR-215-5p up-regulation in gliomas is associated with poor prognosis [31, 32]. Consistently, we also find that miR-215-5p is up-regulated in gliomas and miR-215-5p level is higher in high-grade gliomas. Moreover, we demonstrate that miR-215-5p promotes cell proliferation, clone formation, migration and suppresses apoptosis of gliomas by down-regulating PCDH9 expression. Thus, our study provides functional evidence which fully supports that miR-215-5p is a prognostic factor for gliomas.

A novel finding of our study is that we identify PCDH9 as a direct target of miR-215-5p. We performed a stringent integrative analysis and found that miR-215-5p is one of the few candidate miRNAs which were predicted to target the 3′ UTR of PCDH9 by miRDB and TargetScan and the promoter of PCDH9 by microPIR database. Then, we confirmed the association between miR-215-5p up-regulation and PCDH9 down-regulation in glioma samples and cell lines. The luciferase result that miR-215-5p targets the promoter and 3′ UTR of PCDH9 at the same time suggests that miR-215-5p inhibits PCDH9 expression at the transcriptional and posttranscriptional levels. Indeed, using PCDH9-HA vector in which PCDH9 CDS is under the control of its own promoter and 3′UTR, we demonstrated that synergetic suppression of miR-215-5p on PCDH9 expression is more efficient than targeting the promoter or 3′UTR alone. This kind of dual inhibition is very rare and similar finding is discovered in hepatoma cell lines recently. Our results confirm and extend the generality of the dual inhibition of target genes by miRNAs.

It’s common to see that predicted miRNAs for a target gene from different databases is quite different. Our results suggest that, besides interaction with 3′UTR, additional interaction with the promoter of the same target gene might be a bonus for the microRNA-mediated inhibition. As the miRNA-based therapies are on the horizon [33, 34], a few issues remain to be addressed, including the efficacy and off-target effect [35]. On this occasion, targeting the miRNAs with dual inhibitory effects might achieve high efficacy and avoid off-target effects.

In summary, we find that synergetic suppression of miR-215-5p on PCDH9 expression is more efficient than targeting the promoter or 3′UTR alone in gliomas, and miR-215-5p promotes aggressive glioma phenotypes via inhibiting PCDH9 expression. Our study provides a novel mechanism for the PCDH9 dwon-regulation and suggests that miR-215-5p might be a therapeutic target in gliomas.

## MATERIALS AND METHODS

### Clinical samples

The study was approved by the Review Boards of Wuxi Second People’s Hospital (Wuxi, China) and conducted according to the principles expressed in the Declaration of Helsinki. Written informed consent was obtained from each patient. Thirty primary glioma tissue samples were collected in Wuxi Second People’s Hospital. Eight normal brain tissue samples were obtained from non-glioma patients undergoing brain surgery. All cases were confirmed the pathological diagnosis. Grading of gliomas was performed according to the 2007 World Health Organization Classification criteria. In this study, all the cases of glioma were classified as low grade (WHO I and II) or high grade (WHO III and IV) for statistical analysis. Tissues were freshly resected during surgery and immediately frozen in liquid nitrogen for subsequent total RNA extraction. RNA was extracted from tissues and cell lines using TRIzol reagent (Invitrogen, USA).

### Cell cultures

HEK293 cell line, glioma cell lines U87 and U251 from ATCC were maintained in Dulbecco’s Modified Essential Medium (DMEM) with 10% fetal bovine serum (FBS), 100 U/ml penicillin and 100 mg/ml streptomycin. Cells were cultured in a humidified atmosphere with 5 % CO2 at 37°C.

### Cell proliferation assay

Cell lines transfected with miRNA mimic or inhibitor and PCDH9 vector were seeded into a 96-well plate in triplicate at the concentration of 4×10^3^ cells per well. The cell growth was measured by 3-(4, 5-dimethylthiazol-2-yl)- 2, 5-diphenyltetrazolium (MTT) bromide assay at day 2, 4, 6, 8 and 10, respectively. Cells were incubated with 5 mg/ml MTT for 4 h, and subsequently solubilized in DMSO (100 ul/well). The absorbance at 570 nm was then measured using an ELISA reader.

### Colony formation assay

Cells lines transfected with miRNA mimic or inhibitor and PCDH9 vector were plated in duplicate in a 6-well plate. After incubation at 37°C for 14 days, the colonies were stained with Crystal Violet solution in methanol for 15 min. Colonies > 50 um in diameter were counted under a microscope. The data were presented as fold change relative to the control group.

### *In-vitro* cell migration assay

The cell lines transfected with miRNA mimic or inhibitor and PCDH9 vector were trypsinized and resuspended as a single-cell suspension. A total of 1×10^5^ cells in 0.5 mL serum-free DMEM were seeded onto 8-um pore polycarbonate membrane Boyden chambers inserted in a transwell apparatus (Costar, Cambridge, MA). Then, 600 ul DMEM with 10 % FBS was added to the lower chamber. After incubation for 24 h at 37°C, the cells on the top surface of the insert were removed and the cells that migrated to the bottom surface of the insert were fixed in 100 % methanol and stained with 0.5 % crystal violet. The number of migrated cells was counted under a microscope and the data were presented as fold change relative to the control group.

### Real-time PCR

To detect the relative levels of miRNAs and PCDH9, quantitative real time-PCR (qPCR) was performed. Briefly, Total RNA was isolated from tissues or cell lines using TRIzol reagent, according the manufacture’s protocol. For PCDH9, the cDNA was generated with 1 ug total RNA using reverse transcription using MMLV reverse transcriptases (Promega) and random primers. Actin was used as an endogenous control. qRT-PCR primers were as follows. PCDH9: AGGAACTCCCTTTGGACAACACCT (forward) and TGCACTCTGAGGCACTGAAGT GAT (reverse); actin: ACCAACTGGGACGACATGGAGAAA (forward) and TAGCACAGCCTGGATAGCAA CGTA (reverse). For mature miRNA quantification, cDNA was synthesized using specific stem-loop universal primers (60 ng) and a TaqMan microRNA reverse transcription kit. U6 small nuclear RNA was used as an internal control. The reaction condition was as follows: 30°C for 10 min; 42°C for 1 h; 85°C for 5 min; 5°C for 5 min. TaqMan miRNA assays miR-215-5p (4426961) and U6 snRNA (001973) were from Applied Biosystems. The qPCR conditions were 95°C for 2 min followed by 40 cycles of 95°C for 15 s and 60°C for 30 s. The fold change for each target gene relative to the control group was calculated using the ΔΔCt method. The miR-215-5p mimic, inhibitor and control were from Ambion.

### Plasmid construction

The 500bp 3′-UTR sequence of the human PCDH9 containing miR-215-5p binding site was amplified by PCR and cloned into pMIR-report vector. The 500bp promoter of the human PCDH9 containing miR-215-5p binding site was amplified by PCR and cloned into pGL4.10 vector. The full-length protein coding sequence of PCDH9 was amplified from human brain tissues. The promoter, CDS and 3′UTR was cloned into pcDNA3.1 vector to ensure that PCDH9 CDS is under the control of its promoter and 3′UTR. Cell lines were transfected with the above vectors using the Lipofectamine 2000 transfection reagent (Invitrogen) according to the manufacturer’s protocol.

### Luciferase activity assay

For luciferase reporter assays, HEK293 cells were transiently transfected with pMIR-report containing PCDH9 3′-UTR or pGL4.10 containing PCDH9 promoter, miR-215-5p mimic or inhibitor using Lipofectamine 2000. After 48h, reporter gene activity was measured by the dual-luciferase assay-system (Promega). Renilla luciferase activity was used to normalize for transfection efficiency. The data were presented as fold change relative to the control group.

### Apoptosis assay

Cells lines were transfected with miR-215-5p mimics or control and PCDH9 vector for 2 days. For Annexin V-FITC Apoptosis assay, cells were trypsinized, washed, and stained with Annexin V-FITC Apoptosis kit (abcam, ab14085) in the dark for 15 min at room temperature. Then, the stained cells were analyzed by MoFlo XDP (Beckman Coulter, Inc). For TUNNEL assay, cells were fixed with 4% PFA, permeated with 0.25% Triton®X-100, and stained with Click-iT® TUNEL Alexa Fluor® Imaging Assay kit (C10245). Then, the stained cells were analyzed by fluorescence microscope.

### Western blot

Proteins were extracted from cell lines using sodium dodecyl sulfate lysis buffer (2% sodium dodecyl sulfate, 10% glycerol, 0.1 mM dithiothreitol, and 0.2 M Tris–HCl, pH 6.8). Protein samples (50 ug) were resolved by SDS–PAGE and analyzed by immunoblots. PCDH9 antibody was from abcam (1:1000, ab117115).

### Statistical analysis

Statistical analysis was performed using GraphPad Prism software. All data were presented as mean ± SEM. Statistical analysis was performed by two-tailed Student t test for two groups and one way ANOVA with Newman-Keuls post hoc test for more than two groups. Bivariate correlations between the study variables were calculated using Pearson’s correlation coefficients. Statistically significant differences were defined as P < 0.05. For all, *P<0.05, **P<0.01, ***P<0.001.
